# Microbial perspective on the skin–gut axis and atopic dermatitis

**DOI:** 10.1515/biol-2022-0782

**Published:** 2024-04-13

**Authors:** Bo Qu, Xue-er Zhang, Haoyue Feng, Bonan Yan, Yingchun Bai, Shanlin Liu, Yuhua He

**Affiliations:** Hospital of Chengdu University of Traditional Chinese Medicine, No. 39 Shi-er-qiao Road, Chengdu, 610072, Sichuan Province, P.R. China; Chengdu University of Traditional Chinese Medicine, No. 37 Shi-er-qiao Road, Chengdu, 610072, Sichuan Province, P.R. China

**Keywords:** atopic dermatitis, microbiome, genomics, gut, skin axis, immunology

## Abstract

Atopic dermatitis (AD) is a relapsing inflammatory skin condition that has become a global health issue with complex etiology and mounting prevalence. The association of AD with skin and gut microbiota has been revealed by virtue of the continuous development of sequencing technology and genomics analysis. Also, the gut–brain–skin axis and its mutual crosstalk mechanisms have been gradually verified. Accordingly, the microbiota–skin–gut axis also plays an important role in allergic skin inflammation. Herein, we reviewed the relationship between the microbiota–skin–gut axis and AD, explored the underlying signaling molecules and potential pathways, and focused on the potential mechanisms of probiotics, antimicrobial peptides (AMPs), coagulase-negative staphylococci transplantation, fecal microbiota transplantation, AMPs, and addition of essential fatty acids in alleviating AD, with the aim to provide a new perspective for targeting microbiota in the treatment of allergic skin inflammation.

## 1 Introduction

Atopic dermatitis (AD) is a chronic relapsing inflammatory skin disease mainly manifested as persistent itching of the skin clinically, which predominantly occurs in infants and adolescents but may also affect adults or the elderly. The incidence of AD shows an upward trend in recent decades, impacting approximately 15–20% of children and 1–3% of adults worldwide [1]. Although the pathophysiology of AD has not been completely understood, numerous studies have demonstrated that skin barrier disruption, serine protease disruption, keratinized desmosomes, massive secretion of inflammatory factors, and CD4^+^ T cell activation contribute to the onset of AD. Among them, T cell-mediated immune responses (Th1/Th2 imbalance and Thl7/Treg imbalance) may play a dominant role [2].

The application of emerging techniques, such as meta-transcriptomics, metagenomics, and metabolomics, has confirmed that local microorganisms can influence immune function at distal sites. For example, the gut microbiota can alter the skin phenotype through immunomodulatory effects, leading to the generation of terms such as the skin–gut axis and the microbiota–skin–gut axis. This article reviews the relationship between the microbiota–skin–gut axis and AD from the perspective of skin and gut microbiota, as well as their interacting signaling molecules and potential pathways, and attempts to elucidate the important roles of these pathways in the pathological mechanisms of AD. In addition, this review focuses on the potential mechanisms of microbe-related treatments such as probiotics, microbiota transplantation, and antimicrobial peptides (AMPs) in alleviating AD.

## 2 Microbial characteristics in AD

There is a complex ecosystem of microbiota that exist inside and outside the human body, known as human microbial communities, consisting of thousands of microbial species and trillions of microbial cells. Culture-based approaches have traditionally been used for the study of bacterial species, but many bacterial strains cannot be cultured in the laboratory, and bacterial culture-dependent methods under standard laboratory conditions can merely detect less than 1% of bacterial species [3]. Due to the harsh culture conditions for bacteria, the actual species of bacteria existing in the microbiome are often underestimated. The continuous development of molecular biology and bioinformatics has made significant contributions to microbiome research. At present, 16S rRNA gene sequencing, metabolism, and metagenomics are the most commonly used methods for microbial species research. 16S rRNA gene sequencing has allowed species-level and even strain-level resolution of the microbiome. Metagenomics provides a means of assessing the total genetic pool of the microbiome, in a culture-independent manner [4]. In recent years, researchers have regarded microbiota as a complex human trait and used quantitative and statistical genetic approaches to describe the genetic architecture of the microbiome in association with different host genomic loci. These modern molecular and bioinformatic techniques contribute to defining the genetic diversity of microorganisms and clarifying their relationship with commensal and pathogenic microbiomes.

### 2.1 Overview of skin microbiota in AD

The skin is a complex and dynamic ecosystem inhabited by millions of bacteria, fungi, and viruses. These microbes are collectively referred to as the skin microbiota, which is critical for protecting the skin from pathogens. The bacterial populations on the skin often adapt to the niche they occupy in the skin based on the unique physicochemical properties of the skin surface (temperature, age, sebum content, sweat, etc.) [5]. In accordance with existing literature, regions of the body that are abundant in sebum tend to be primarily colonized by lipokeratinocytes (Propionibacterium) and fungi (Malassezia). Conversely, areas that are moist, such as the axilla, are predominantly inhabited by *Corynebacterium* spp. and *Staphylococcus* spp. On the other hand, dry regions like the flexed side of the limbs exhibit varying populations consisting of β-Proteobacteria, Flavobacteria, and Malassezia [6]. The skin microbiota has the ability to influence the immunological response of the host by regulating several factors such as keratinocytes, AMPs, lipid antimicrobials, and cytokines. These regulatory mechanisms contribute to the enhancement of the skin barrier function, ultimately leading to the maintenance of homeostasis [7].

Disruption of the homeostasis of the internal environment in AD patients disrupts the microbial ecology of the skin, which is mainly characterized by significant changes in microbial diversity, number, composition, and metabolism [8]. According to the hygiene hypothesis, there is an inverse relationship between AD and early life exposure to microorganisms [9]. The earliest exposure of infants to microorganisms begins at delivery, and the mode of delivery exerts a significant impact on the newborn’s skin microbial community. For instance, the microbial community of infants delivered through the vagina is dominated by *Lactobacilli*, whereas that of infants delivered through cesarean section is dominated by *Staphylococcus*, *Corynebacterium*, and *Propionibacterium* [10]. Thus, multiple factors such as the mode of birth, stress, diet, and environmental pollution affect the skin microbiota in AD patients, which mediate the host immune response, induce IgE production and Th2 cell activation, and thus cause the exacerbation of AD symptoms [11]. A recent longitudinal study of the skin microbiota in AD patients has found altered microbial diversity on the AD skin surface (Table 1), as evidenced by a decrease in *Streptococcus* spp., *Acinetobacter* spp., *Corynebacterium* spp., and *Prevotella* spp., but an increase in *Staphylococci* such as *Staphylococcus epidermidis* and *haemolyticus* spp., among which the colonization density of *Staphylococcus aureus* is closely related to AD severity [12].

**Table 1:** Characterization of the skin and gut microbiota in AD

Position	Microbial alterations		Immune dysregulation
Skin	↓ *Streptococcus* spp.	↑ *S. epidermidis*	↓ AMPS
	↓ *Acinetobacter* spp.	↑ *Staphylococcus hemolysis*	↑ IL-4, ↑ IL-5,
	↓ *Prevotella* spp.	↑ *Staphylococcus* spp.	↑ IL-13, ↑ IL-22,
	↓ *Corynebacterium*	↑ *S. aureus*	↑ IL-31, ↑ IgE,
	↓ *Propionibacterium acnes*		↑ TSLP
	↓ *Cutibacterium* spp.		
	↓ *Acinetobacter*		
Gut	↓ *Lactobacillus*	↑ *Bacillus coli*	↓ Treg, ↓ IgA,
	↓ *Bifidobacteria*	↑ *C. difficile*	↑ IL-25, ↑ IL-33
		↑ *S. epidermidis*	

Note: ↑: increase; ↓: decrease.

### 2.2 Overview of gut microbiota in AD

The human intestinal tract is home to a large and complex bacterial community, collectively referred to as the gut microbiota [13]. A total of 2,172 gut microbiota species in 12 phyla have been isolated from human individuals, of which 93.5% belong to Bacteroidetes, Firmicutes, Actinobacteria, and Proteobacteria [14]. Recent studies have demonstrated the critical involvement of gut microbiota in the development of allergic diseases such as intestinal inflammatory diseases, asthma, and AD [15]. The metabolites such as amino acid metabolites, short chain fatty acids (SCFAs), and oligosaccharides produced by gut microbiota can form a mucosal layer and constitute the intestinal barrier to avoid translocation of microbiota into the systemic circulation to initiate infection, thereby mediating material metabolism and immune function [16]. The immune balance between the gut microbiota and the host is vulnerable to diet, disease, and other factors. Some food ingredients can disrupt the intestinal barrier and then accelerate pathogenic microbial colonization [17]. Pathogenic microorganisms can enter the blood circulation and activate intestinal mucosal immunity, inducing the production of immune cells and inflammatory cytokines [18].

The imbalance of gut microbiota is also responsible for the pathogenesis of AD. By following high-risk infants for the first year of life and taking stool samples before the onset of atopic symptoms, several studies have found that infants whose mothers have a history of AD have high levels of detectable *Escherichia coli* in their stools, and a decreased ratio of *Bifidobacterium* to *Clostridium difficile*, which directly affects the development of the immune system later in life and increases the risk of developing AD. The gut microbial diversity of AD patients is reduced compared to that of healthy individuals (Table 1), mainly characterized by a significant decrease in the relative abundance of beneficial genera such as *Lactobacillus* and *Bifidobacterium*, but a significant increase in the relative abundance of *E. coli, C. difficile*, and *S. aureus* [19]. Reduced gut microbial diversity and decreased number of propionate and butyrate-producing bacteria are indicative of aggravated AD, while propionate and butyrate, as components of SCFAs, have anti-inflammatory and immunomodulatory effects and are essential for alleviating chronic inflammatory diseases including AD [20].

## 3 Skin–gut axis communication mediated by immune and neuroendocrine systems

**Figure 1:**
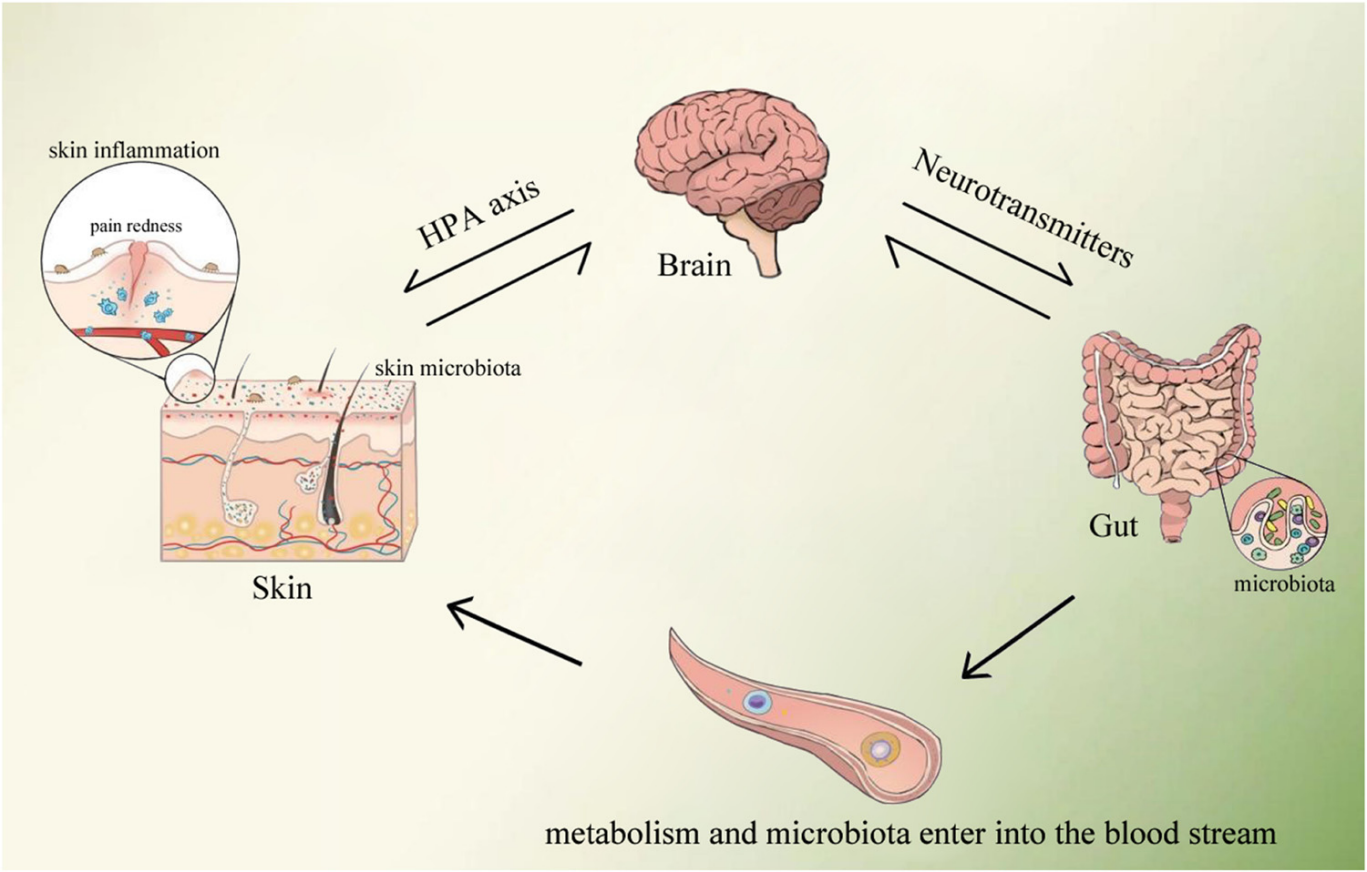
Neuroendocrine-mediated communication in the skin–gut axis. HPA axis: hypothalamic–pituitary–adrenal axis.

There is a two-way relationship between gastrointestinal health and skin homeostasis, which is affected by diet, mood, and many external factors. Scientists have proposed that the skin–gut axis is a part of the gut–brain–skin axis, and the gut, brain, and skin cells are derived from the same germ layer [21]. Both skin and intestinal organs are the keys to immune and neuroendocrine functions, with high similarity in structure and nerve distribution [22]. The host immune and neuroendocrine systems can mediate skin–gut communication. The regulation of the immune system is mainly dominated by gut microbiota, and the immune imbalance is reflected in the skin. Intestinal dysfunction and gut microbiota imbalance may exert an intermediary effect on inflammatory skin diseases (Figure 1).

### 3.1 Immune-mediated communication in the skin–gut axis

#### 3.1.1 Peyer’s patches

Peyer’s patches are parts of gut-associated lymphoid tissue dispersed throughout the intestine, which can synthesize interleukin-10 (IL-10) and induce T helper cell differentiation to participate in the intestinal mucosal immune system. Cytokines or immune cells from Peyer’s patches can be transported to the skin through circulation, thus regulating the immune state of the skin and improving the defense mechanism [23]. Therefore, it is speculated that Peyer’s patches provide a possible connection for gut–skin communication.

#### 3.1.2 Epithelial cells (ECs)

ECs lining the inner surface of the intestinal tract and the outer surface of the skin play a pivotal role in protecting the human body from microbial infections. Thus, ECs establish a barrier between the external environment and the internal milieu. Alternations in gut microbiota may lead to an increase in intestinal permeability when the integrity of the intestinal epithelial barrier is destroyed to a certain level, causing the leaky gut syndrome. The formation of a leaky gut exacerbates the penetration of immunogenic molecules, including dietary antigens, bacterial toxins, and related pathogens such as tumor necrosis factor-alpha (TNF-α) and interferon-gamma (IFN-γ) [24]. Activation of toll-like receptors (TLR) and zonulin signaling to initiate a series of inflammatory responses exacerbates the loss of barrier integrity of intestinal ECs, leading to bacterial translocation and inflammatory responses [25]. Subsequently, these immunogenic molecules can enter the bloodstream and accumulate in the skin, interfering with the epidermal barrier and eventually leading to skin immune response and skin inflammation [26].

#### 3.1.3 Serum 25(OH)D

Serum 25(OH)D is an important immunomodulator that exists in the human body in the biologically inactive form of vitamin D. Vitamin D in macrophages regulates endogenous cathepsin and inhibin synthesis, cytokine release, and the body’s defense against pathogens. Serum 25(OH)D deficiency has been shown to trigger an inflammatory environment and gut microbiota imbalance. Elevated serum 25(OH)D levels induced by ultraviolet B (UVB) exposure are associated with increased alpha and beta diversity of gut microbiota [26]. Vitamin D can affect both innate and adaptive immune cells, thereby inhibiting pro-inflammatory responses [27]. It is also suggested that skin exposure to UVB may affect the activation of intestinal immune cells and the release of immune mediators, which in turn remodel gut microbiota, but the specific mechanisms remain to be elucidated. Vitamin D has antiproliferative, pro-differentiation, anti-apoptotic, and immunomodulatory effects in the skin. Epidemiologic studies have shown that populations living at higher geographic latitudes have lower sun exposure, lower vitamin D production, and increased prevalence of AD [28]. In addition, studies have observed that vitamin D level deficiency leads to an increased likelihood of developing AD and that the severity of AD is negatively correlated with vitamin D levels [29].

### 3.2 Neuroendocrine-mediated communication in the skin–gut axis

#### 3.2.1 Neuropeptides

From the perspective of the neuroendocrine system, the activities of the central nervous system such as depression and anxiety, brain cognitive development, and stress response are related to gut microbiota. Mental stress can accelerate the excessive growth of intestinal microorganisms, increase intestinal permeability, destroy the intestinal immune barrier, and promote the release of neuropeptides from the nervous system [30]. Neuropeptides can stimulate keratinocytes to produce pro-inflammatory cytokines (such as IL-1α, IL-6, and IL-8), enhance the migration, antigen expression, and sensitization of Langerhans cells, induce mast cell degranulation and release of histamine and other mediators, and trigger peripheral blood mononuclear leukocytes to release IFN-γ, IL-4, TNF-α, and IL-10, thus eventually initiating inflammatory skin diseases (such as AD, psoriasis, and rosacea) [31,32].

#### 3.2.2 Secretion of hormone-like compounds

Hormone-like compounds are a variety of metabolites secreted by gut microbiota (such as SCFAs and cortisol) and neurotransmitters (gamma-amino butyric acid [GABA], 5-hydroxytryptamine [5-HT], dopamine, and tryptophan). These hormone-like pleiotropic compounds are released into the blood and act on distant skin [33]. The skin–gut axis has a two-way effect, and changes in skin-related structural components also cause changes in gut microbiota as illustrated in Figure 1.

Both the skin and the gut are key to the immune and neuroendocrine systems, and the two may communicate through (1) the releasing effects of neural signaling molecules and (2) altered characterization of the skin and gut microbiota, and interactions between microbiota metabolites and associated immune cells.

## 4 Pathways of skin and gut microbiota affecting AD

### 4.1 Skin barrier pathways

**Figure 2:**
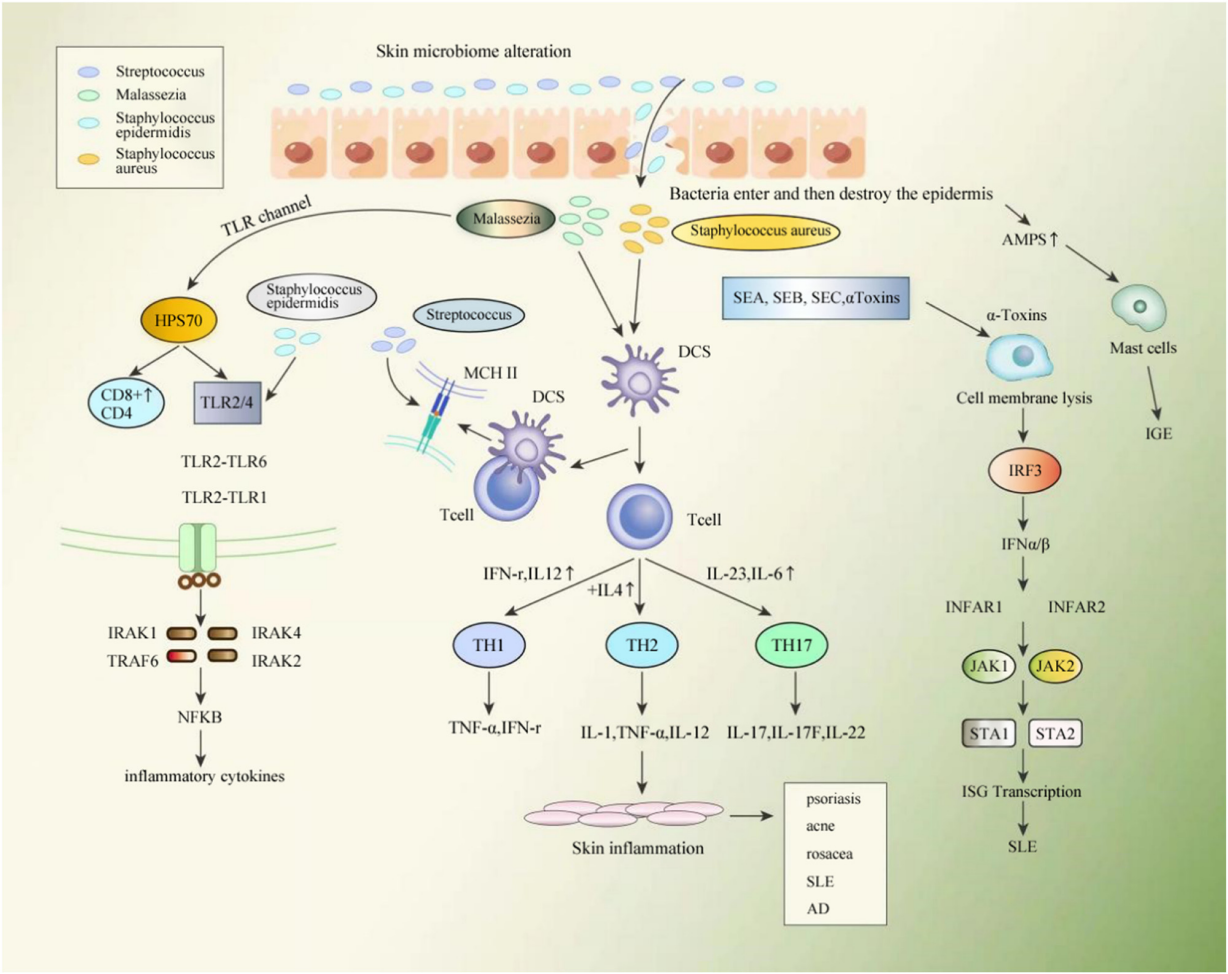
Crosstalk between the skin and the microbiome under conditional pathogenic bacteria. IL: interleukin; TNF-α: tumor necrosis factor-α; IFN-γ: interferon-γ; SCFAs: short chain fatty acids; SEA/B: staphylococcal enterotoxin A/B; TSST: toxic shock syndrome toxin.

The dysbacteriosis of microbiota in the AD skin surface can be attributed to the destruction of skin barrier function, the reduced contents of natural moisturizing factors, the increase in the surface pH value, and the changes in surface lipid compositions [34]. The latest research has shown that the colonization of *S. aureus* is related to the pathogenesis, disease flare, and disease phenotype of AD. *S. aureus* has a variety of highly evolved cell wall proteins and secretory factors, which can adhere to human skin and disturb the skin barrier through physical, chemical, and inflammatory mechanisms (Figure 2). The following aspects are involved.

#### 4.1.1 Epidermal barrier damage

*S. aureus* colonized on the surface of AD skin can express a variety of toxin factors and proteases, damaging the skin barrier and causing superficial and invasive skin infections. For example, *S. aureus* alpha-toxin (also known as pore-forming toxin) can penetrate the host cell membrane and dissolve the cell membrane [35]. Many proteases (serine protease, kallikrein KLK6, 13, and 14) can dissolve the stratum corneum, resulting in impaired skin barrier function [36]. Lipoteichoic acid (LTA), a product of the *S. aureus* cell wall, leads to skin barrier damage by inhibiting the expression of epidermal barrier proteins including filament polyprotein and cucumber protein [37].

#### 4.1.2 Increase of bacterial adhesion

*S. aureus* can produce several surface molecules such as clumping factors A and B (ClfA and ClfB), fibronectin-binding protein (FnBP), and iron-regulated surface determinant A (IsdA) to adhere to the human stratum corneum [38]. In addition, the lack of skin lipids and the release of Th2 cytokines such as IL-4 in AD patients increase the expression of fibronectin and fibrinogen, conferring a favorable environment for *S. aureus* to adhere closely to keratinocytes [39].

#### 4.1.3 Defect of bacterial clearance

The average pH value of the skin of AD patients is slightly alkaline, causing a decrease in the sphingosine level in the stratum corneum [39]. Sphingosine belongs to sphingolipids (SPL). SPL is an important component of cell membrane structure, and pharmacological SPL antagonism exacerbates vascular permeability and inflammation [40]. The instability of the stratum corneum cell membrane affects the transdermal water loss, pH value, serum IgE, and eosinophil of AD, which finally leads to the defect of skin bacterial clearance and accelerates the colonization of pathogenic bacteria.

Microorganisms such as fungi and bacteria cover the skin surface and reside in skin appendages, including hair follicles, sebaceous glands, and sweat glands. Skin resident microorganisms could promote the production of AMPS by keratinocytes and the production of complement by antigen-presenting cells and/or other innate immune mediators, which interact with the host to establish a functional immune response and prevent the overgrowth of pathogenic microorganisms. Skin microbial disorders and bacteria damage the skin barrier, activate downstream molecular signals, and induce chronic inflammatory responses.

(1) Stimulates AMPS to degranulation of mast cells, stimulates dendritic cells to bind to T cells, thereby inducing proliferation and polarization of T cells (Th1/Th2 and Th17/Treg imbalance). (2) Release of toxin factors, including staphylococcal enterotoxin A (SEA), staphylococcal enterotoxin B (SEB), SEC, and α-toxin-induced cell membrane lysis. (3) Hps70 activation through TLR channels leads to NF-κB overexpression, which in turn promotes the release of inflammatory cytokines, triggering cutaneous inflammatory responses.

### 4.2 Immune pathways

Despite the joint involvement of skin and gut microbiota in the human immune system, there are still differences between the two. Gut microbiota modulates gene regulation by regulating intestinal lymphoid structures, controlling stability, and activating the innate immune system [5]. Skin microbiota affects the immune system by producing AMPs, increasing the activity of the complement system, and affecting the expression of functional variants of TLRs and nucleotide-binding oligomerization domain/contain caspase-recruitment domain receptors [41].

#### 4.2.1 Skin microbial immune pathways

Microbiota can trigger pro-inflammatory mechanisms by releasing toxin factors and inflammatory drivers, thereby affecting the expression of TLRs and the function of host immune cells. It mainly involves the following aspects: (1) staphylococcal toxin factor-elicited immune responses: staphylococcal toxin factors are mainly released by the mediation of staphylococcal enterotoxins (SEs), SEA, SEB, and toxic shock syndrome toxin (TSST)-1. These toxins function as superantigens (SAgs) [42] to activate T cell clones, resulting in substances capable of mounting an extremely strong immune response, which subsequently triggers IgE-mediated mast cell degranulation and elevates Th2 cytokines [43]; moreover, they bind to human leukocyte antigen-DR on Langerhans cells and macrophages to stimulate the production of IL-1, TNF-α, and IL-12, thus contributing to the exacerbation and recurrence of AD [43]. (2) Secretion of thymic stromal lymphopoietin (TSLP): *S. aureus* is also able to trigger keratinocyte apoptosis, releasing TSLP. TSLP release can mediate the itch response by inducing skin dendritic cell activation, promoting IL-4 and IL-13 secretion, and influencing Th2 cell recruitment [36]. (3) *Malassezia*-stimulated inflammatory factors: *Malassezia* colonized on AD skin mediates dendritic cell maturation and stimulates keratinocytes to produce a variety of inflammatory cytokines (e.g., IL-4, IL-6, IL-8, and TNF-α); *Malassezia* also induces IgE-mediated mast cell degranulation to release leukotrienes, thus perpetuating inflammation [44]. However, *Malassezia* does not produce IL-12. IL-12 is a determinant of Th1-type immune responses that can promote the production of Th1 class factors and inhibit the production of Th2 class factors, thereby inhibiting Th2-type immune responses. Hence, *Malassezia* favors the induction of Th2-type immune responses in AD pathogenesis [45].

#### 4.2.2 Gut microbial immune pathways

In addition to intestinal lymphoid structure, gut microbiota may also regulate intestinal immune homeostasis and cause peripheral inflammation through the intestinal vasculature. Several lines of evidence have suggested that gut microbiota activates TLRs 2, 6, and 4 and causes inflammatory responses by activating the canonical NF-κB pathway while upregulating vascular endothelial growth factor receptor 2 to promote intestinal angiogenesis [46]. Interestingly, intestinal vascular endothelial growth factor C (VEGF-C) can recognize microorganisms through TLR complexes. Therefore, VEGF-C may be a key downstream effector of gut microbiota, regulating the recruitment of macrophages and Tregs and the balance of IL-9/IL-17 [47]. An assessment of gut microbiota and innate immune responses in IgE-associated eczema has revealed that the number of *Ruminococci* in the stools of children with AD is lower than that of healthy controls, and *Ruminococci* has a negative correlation with TLR2-induced IL-6 and TNF-α, while *Enterobacteriaceae* is positively correlated with TLR4-induced TNF-α [48]. *Bifidobacteria* can directly or indirectly enhance the immunosuppressive function of Tregs by stimulating the IL-10/IL10Ra signaling loop, indicating that the number of *Bifidobacteria* in the gut affects the occurrence of AD [49]. *Lactobacillus plantarum* LM1004 reduces Th2 and Th17 cell transcription factors, serum IgE, and TSLP, but increases the transcription factors of Tregs, Th1 cells, and filaggrin, while butyrate, which modulates gut microbiota, significantly improves AD symptoms [50]. The decreased IL-17A expression has been found in plasma samples of AD children in association with a decrease in *Romboutsia* and *Dorea* and an increase in *anaerobe* and *Faecalibacterium prausnitzii* in the gut [51].

### 4.3 Metabolite pathways

Microbiota can secrete a variety of metabolites such as SCFAs, cortisol, GABA, 5-HT, dopamine, and tryptophan. These metabolites can accumulate under the skin or be released into the blood, affecting keratinocyte differentiation, skin hydration ability, and epidermal barrier function by downregulating the expression of keratin and acting on the skin immune system. The microbial metabolites in the skin–gut axis are closely related to the pathogenesis of AD, and different metabolites can be independent factors of the clinical phenotype and recurrence of AD.

#### 4.3.1 Polyunsaturated free fatty acids (PUFAs)

PUFAs are divided into ω-6PUFAs and ω-3PUFAs. Arachidonic acid (ARA) in ω-6PUFAs is generally considered to induce and promote inflammation. *Malassezia* colonized on AD skin can produce a variety of enzymes (including lipase and phospholipase), which release PUFAs from sebum lipids to trigger inflammation [52]. This process may be closely related to the release of ARA. ARA can produce pro-inflammatory eicosanoid acids (prostaglandins) and leukotrienes under the action of phospholipase A2 (PLA2), resulting in inflammation and stratum corneum damage. It has been found that mice overexpressing PLA2III exhibit histologically similar skin inflammation to AD, such as hyperkeratosis, acanthosis, incomplete keratosis, erosion, ulcers, and sebaceous hyperplasia [53]. Interestingly, octadecenoic acid and ARA isomers can be detected in the T lymphocyte membrane of AD patients [54]. Therefore, *Malassezia* colonized on AD skin may affect the formation of trans-fatty acid isomers in the T cell membrane, the release of pro-inflammatory mediators, and the activity of T cell signaling transduction, eventually leading to the pathogenesis of AD.

#### 4.3.2 SCFAs

SCFAs, including butyrate, acetate, and propionate, are produced during bacterial fermentation of undigested polysaccharides, which can reduce the permeability of the intestinal barrier, enter the blood, and act on the immune system. SCFAs mainly act on the immune system through two mechanisms to elicit systemic effects, namely, activating the immune signaling through G protein-coupled receptors (GPR41/GPR43/GPR109A) and inhibiting histone deacetylases [55]. For example, propionate can promote the generation of macrophage and dendritic cell precursors, hinder the differentiation of Naïve T cells into Th2 cells, and thereby prevent the development of allergic inflammation [56]. SCFAs may inhibit skin inflammation by increasing the number of skin resident Tregs [57]. Topical administration of SCFAs has been demonstrated to restore immune function and control inflammatory skin diseases potentially through the modulation of T cells, which represents a novel strategy for protocols using bacterial metabolites to control skin inflammation.

#### 4.3.3 Tryptophan

The ligands of tryptophan metabolites can signal through the aryl hydrocarbon receptor (AhR) to inhibit the production of inflammatory cytokines in the skin and gut, thereby reducing the occurrence of inflammatory reactions [58]. Indoles and their derivatives, such as 3-indoleacrylicacid (IA), 3-indoleacetic acid (AA), indole-3-carboxaldehyde (I3C), and indole acetaldehyde (IAId), belong to tryptophan metabolites. (1) IAId, a product of tryptophan metabolism by skin microbes, can inhibit the production of TSLP in keratinocytes in an AhR-dependent manner, thus reducing skin inflammation in mice with AD-like dermatitis. (2) I3C, a tryptophan metabolite of *Bifidobacterium longum* CCFM1029, can activate the AhR-mediated immune signaling pathway to improve AD symptoms [59]. (3) Kynurenic acid (KYNA), a product of tryptophan metabolism of *Bifidobacterium* LKM512, has mucosal protection and immunomodulatory effects, which can relieve AD pruritus and improve the quality of life of AD patients [60].

#### 4.3.4 Polyamines

Polyamines are metabolites produced by a variety of bacteria that exist widely in nearly all living organisms. The function of the intestinal mucosal barrier in healthy adults to prevent antigen invasion is mainly maintained by the concentration of polyamines secreted by gut microbiota. Polyamines alleviate systemic inflammation by inhibiting the synthesis of inflammatory cytokines. Intake of the probiotic strain LKM512 facilitates the production of polyamines and butyrates in the gut of AD patients, induces Th1 cytokine production, and promotes intestinal mucosal barrier function recovery, thus alleviating AD symptoms [61]. In contrast, reduced concentrations of intestinal polyamines have been detected in adults with refractory AD [62]. Therefore, the decrease of polyamines may be associated with the recurrence of AD, but more studies are still needed to verify the relationship between polyamines and AD.

The microbial metabolites in the skin–gut axis are closely associated with the pathogenesis of AD, and different metabolites can be independent factors for the phenotype and recurrence of AD. The current AhR-dependent anti-inflammatory active agent (Tapinarof) has been shown to reduce inflammatory cytokine production and improve skin inflammatory symptoms in animal models, and its efficacy and safety in treating mild to severe AD have been confirmed in randomized controlled trials [63]. It is suggested that topical administration of microbial metabolites may alleviate inflammatory skin diseases, but the underlying mechanisms remain to be further confirmed.

### 4.4 Neuroendocrine pathways

Emerging evidence has revealed that gut microbiota mediates the effects of emotional and neurological states on the skin, indicating that microbiota is an important factor in the communication network of the skin–gut axis [64]. Microbiota can directly or indirectly regulate the skin–gut axis via neuroimmune and neuroendocrine pathways. This effect is mainly dependent on the interaction between microbiota and the central nervous system and the conduction of microbial signaling molecules. Among them, signaling molecules mainly include metabolites produced by microorganisms, neurotransmitters, and cytokines released during microbial immune responses, etc.

#### 4.4.1 Interactions between the microbiota and the central nervous system

The central nervous system regulates the gastrointestinal system and enteric nervous system via the autonomic nervous system (ANS) (sympathetic and parasympathetic/vagal) and the hypothalamic–pituitary–adrenal (HPA) axis. The central nervous system can directly alter the environment of gut microbiota or indirectly affect gut microbiota through a large number of signaling molecules [65]. ANS-mediated mucus secretion may have a pivotal impact on the intestinal mucus layer, an important habitat for gut microbiota. The ANS can also respond to bacteria containing AMPs by modulating intestinal immune cells such as macrophages and mast cells [66]. Gut microbiota can also produce neuroactive molecules and neurotransmitters, which in turn influence the function of the vagus nerve. Vagotomy can abolish anxiety-like behavior caused by *B. longum* [67]. Pressure alopecia and neurodermatitis are significantly alleviated in mice fed with *Lactobacilli*.

#### 4.4.2 Conduction of microbial signaling molecules

The nervous system is involved in regulating the immune response in the skin of AD patients, forming a neuro-immune-endocrine regulatory network, which is bi-directionally regulated by various biochemical mediators [68]. Studies have shown that AD patients can stimulate skin immune responses by releasing neuropeptides and vasoactive intestinal peptides that regulate the function of keratinocytes and other immune cells [68]. For example, vasoactive intestinal peptides are associated with itch intensity in AD, affect IFN-γ and IL-4 cytokine release, and increase the risk of IgE-mediated mycobacterial sensitization in AD, all of which suggest that neuropeptides may be used as better alternative biomarkers for AD [69]. The conduction elements of molecules are as follows.

##### 4.4.2.1 Metabolites

SCFAs, cortisol, and tryptophan are common metabolites produced by gut microbiota, which can signal to the host through neuroendocrine pathways and receptors on local cells in the gut, implying the potential role of metabolites in the communication between the microbiota and the central nervous system. HPA axis dysfunction is commonly seen in AD patients. The acute reduction in the excitability of the HPA axis increases cortisol levels, resulting in the imbalance of Th1/Th2 and the secretion of cytokines that influence the inflammatory immune phenotype of AD patients. Tryptophan, as a serotonin synthetic precursor in the brain, can affect central serotonin concentrations and downstream neuroactive metabolites, thus directly mediating AD-independent itch responses. SCFAs (butyrate, acetate, and propionate) can stimulate the sympathetic nervous system to interact with neuronal cells and upregulate the expression of neurokines, thereby affecting the skin lesion phenotype of AD [70].

##### 4.4.2.2 Neurotransmitters

Gut microbiota can produce neurotransmitters such as 5-HT and GABA to influence gut–brain communication [71]. Neurotransmitters may be key regulators of the skin–gut axis. Among them, 5-HT is involved in the communication between the immune system and the central nervous system. Peripheral 5-HT has been identified as a potent itch inducer that directly mediates AD-independent itch, which may be related to the expression of specific 5-HT receptors (HTR7). The coupling of HTR7 to the stimulatory receptor TRPA1 triggers neuronal excitation and mediates itch responses [72]. GABA, an inhibitory neurotransmitter mainly produced by lactic acid bacteria and *Bifidobacterium*, can inhibit the production of iNOS, IL-1, and TNF-α to exert anti-inflammatory activity [73]. Recent studies have shown that increased GABA ligands and GABAA receptors may mediate itch responses in inflammatory skin diseases [74]. All these findings suggest that neurotransmitters serve as novel immunomodulators for the treatment of AD.

##### 4.4.2.3 Cytokines released during microbial immune responses

Cytokines are released during microbial immune responses and mediate host responses through neuroimmune pathways [75]. For example, *S. aureus* mediates mast cell degranulation by releasing SAgs, causes an increase in Th2 immunoregulatory factors, and releases TSLP by promoting keratinocyte apoptosis. These immune cytokines may mediate AD itch responses by generating itch signaling via the vagal or spinal neural pathways. Among them, mast cells communicate with the HPA axis through mediators such as tryptophanase, histamine, and neuropeptides. Concomitantly, Mas-related G-protein-coupled receptor is activated to regulate transient receptor channel potential cation channel ankyrin subtype 1 (TRPA1) expression and mediate histamine-independent itch responses [76]. TSLP can be induced to activate neurons and directly stimulate itch sensory nerve fibers, thus producing itch. IL-31 can directly act on a subpopulation of IL-31RA/transient receptor potential cation channel vanilloid subtype 1 (TRPV1)/TRPA1 + dorsal root ganglia neurons [77]. Moreover, IL-31 binds to IL-31RA, activates the JAK-STAT pathway, and thereby mediates itch responses [78]. In a phase II trial, nemolizumab, an anti-IL-31RA monoclonal antibody, significantly alleviates pruritus in patients with moderate to severe AD [79]. Therefore, interventions targeting immune cytokines released by specific microbes to block the communication with neural pathways may provide novel targets for the treatment of skin diseases.

**Figure 3:**
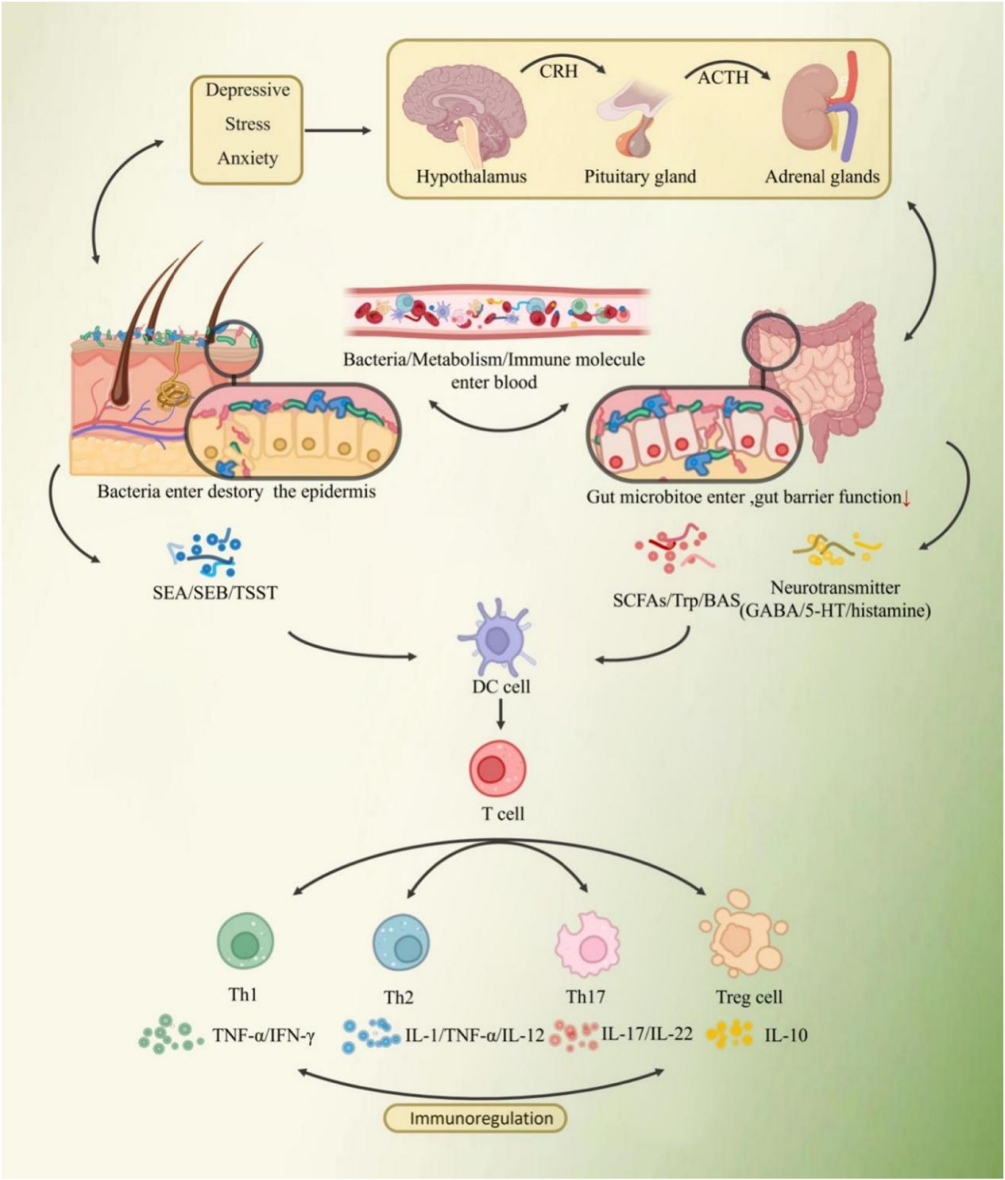
Communication on the “microbe–skin–gut” axis. IL: interleukin; 5-HT: 5-hydroxytryptamine; TNF-α: tumor necrosis factor-α; IFN-γ: interferon-γ; SCFAs: short chain fatty acids; SEA/B: staphylococcal enterotoxin A/B; TSST: toxic shock syndrome toxin; GABA: gamma-amino butyric acid; BAS: basophils; Trp: tryptophan; SCFAs: short chain fatty acids.

In summary, the “microbial–skin–gut” axis affects and exacerbates AD through specific pathways such as the skin barrier pathway, immune pathway, metabolic pathway, and neuroendocrine pathway (Figure 3). The main manifestations are microorganisms adhere to human skin and interfere with the skin barrier function through physical, chemical, and inflammatory mechanisms; release toxins such as SAg and secrete metabolites such as PUAF and dopamine, which cause T-cell proliferation and differentiation, and mediate the Th1/Th2/Th17 immune response, thus participating in AD pathogenesis and influencing AD disease phenotype; and release neurotransmitters such as GABA, 5-HT, or neuroactive metabolites such as SCFAs, cortisol, and tryptophan, which generate pruritic signals and mediate the AD itching response.

The “microbial–skin–gut” axis affects and exacerbates AD mainly through specific pathways such as skin. The “microbial–skin–gut” axis affects and exacerbates AD through specific pathways such as the skin barrier pathway, immune pathway, metabolic pathway, and neuroendocrine pathway.

## 5 AD therapy based on skin and intestinal microorganisms

Currently, the prevalent treatments for AD mainly include topical application of moisturizers, corticosteroids, and anti-inflammatory agents (such as calcineurin inhibitors and phosphodiesterase inhibitors) to control the disease symptom’s severity and duration. Phototherapy and systemic immunosuppressants (Omazumab IGE inhibitors, Fezakinumab unzanumab IL-22 inhibitors, Suchizumab IL-17A inhibitors, and Nemolizumab IL-31 inhibitors) are used for refractory and severe AD by improving skin hydration and repairing skin barrier function, and the above agents target different pathways in the immune system (JAK-STAT, phosphodiesterase 4, AhR receptor, and Th2 cytokines). Exploring the pathogenesis of AD from the microbiota–skin–gut axis and determining the possible communication pathways of microbiota in the axis and the possible mechanism of their effects on AD will provide a new targeted approach for the treatment of AD.

### 5.1 Ingestion of probiotics

To repair the skin barrier by ingesting probiotics and prebiotics has been gradually recognized. Probiotics possess a variety of therapeutic effects: (1) modulating gut microbiota, stimulating mucus secretion, maintaining epithelial tight junction function, and thereby repairing intestinal mucosal barrier integrity; (2) secreting a variety of metabolites and neurotransmitters (SCFAs, phenolics, 5-HT, tryptophan, etc.) that alter intestinal mucosal permeability, followed by reducing the entry of noxious substances into the circulatory system to affect skin barrier function; and (3) inducing the production of the anti-inflammatory cytokine IL-10, stimulating hypothalamic hormone secretion, and thereby modulating skin immune status [80]. In a randomized controlled clinical study, oral *Lactobacillus parasarum* strains can reduce skin sensitivity and increase barrier function. Intake of probiotic strain LKM512 alleviates the symptoms of AD by inhibiting the production of Th1 cytokines and increasing the concentration of polyamines, which may be due to the fact that KYNA in polyamine metabolism can be transported to the brain through the blood to relieve itching [52].

Recent studies have found a strong association between gluten diet and skin diseases. Gluten, a protein found in wheat, rye, and barley, causes immune reactions in patients with celiac disease (CD) and induces cutaneous manifestations such as dermatitis herpetiformis [81]. The incidence of AD in patients complicated with CD is three times higher than that in patients complicated with other allergic diseases [82]. Gluten sensitivity is related to gut microbiota imbalance. Some probiotics (*Bifidobacterium breve, B. longum, Bifidobacterium infantas, L. plantarum, Lactobacillus acidophilus, Lactobacillus casei*, etc.) can hydrolyze gluten polypeptides, decrease TNF-α, and increase IL-10, thereby exerting an anti-inflammatory effect in the intestinal mucosa [83]. Therefore, probiotics may be a promising treatment approach for gluten sensitivity-related AD, especially in AD patients with CD symptoms, but the specific mechanism remains to be further discovered.

### 5.2 Addition of AMPs

AMPs are short peptides with a broad spectrum of antibiotic activities, which modulate the innate immune system of the host to promote pathogen clearance. The production of AMPs such as beta-defensin-2, beta-defensin-3, and LL-37 in AD patients is reduced, which may be attributed to *S. aureus* colonization [46]. The relative reduction of AMPs then exacerbates the colonization of pathogenic microorganisms, leading to a higher risk of developing severe skin, multi-organ infection, and systemic infection in AD patients. Omiganan (OMN) is an AMP with extensive antibacterial and anti-biofilm activities. The use of OMN can effectively restore the ecological balance of the skin in adult patients with moderate to severe AD, reduce *S. aureus* colonization, and increase microbial diversity indexes [84]. OMN strongly drives the interferon regulatory factor signaling induced by TLR and the NF-κB inflammatory pathway, increases the levels of II-IFNɣ secreted by T cells, and thereby achieves anti-inflammatory and anti-viral effects [85]. Thus, AMPs may reduce skin inflammation and repair epidermal barrier function by modulating the innate immune system. This also further confirms that the role of AMPs in both pro-inflammatory and anti-inflammatory pathways relies on their immunomodulatory properties rather than antimicrobial properties. The pathways to reduce the colonization of pathogenic microorganisms not only depend on the use of traditional antibiotics but may also rely on antibacterial effects by reducing the production of pro-inflammatory factors.

### 5.3 Microflora transplantation

#### 5.3.1 Coagulase-negative staphylococci (CoNS) transplantation

CoNS are skin commensal bacteria found in *Staphylococcus* species, including *S. epidermidis, Staphylococcus hominis, S. haemolyticus, Staphylococcus capitis*, and other common isolates. CoNS residing in the skin can compete against invading pathogens by producing AMPs or increasing the activity of the complement system (IL-1 levels) to limit the colonization of pathogens, achieving an anti-colonization effect. The antimicrobial capacity of CoNS is strongly associated with *S. aureus* colonization, mainly with *S. epidermidis* and *S. hominis* [86]. For example, *Staphylococcus lugdunensis*, a species of CoNS, has been shown to produce a polypeptide antibiotic to inhibit *S. aureus* in a rat nasal colonization model [87]. The phenol-soluble modulin peptide expressed by *S. epidermidis* is also able to kill *S. aureus* [88]. *Staphylococcus muciniphila* transplanted into healthy humans can inhibit *S. aureus* via lysophosphatidylcholine and activate the innate immune system to effectively alleviate AD [89]. CoNS transplantation may produce antibiotic-like compounds that antagonize competing pathogens to effectively alleviate AD. CoNS transplantation has a stronger ability to restore the homeostasis of skin microbiota than antibiotic therapy because the nonspecific antimicrobial effects of drug-derived antibiotics are likely to kill protective strains such as cons and instead increase the possibility of *S. aureus* colonization.

#### 5.3.2 Fecal microbiota transplantation (FMT)

FMT, where fecal microbiota from a healthy donor is transplanted into a patient’s gastrointestinal tract, is currently being explored as a potential therapy to reconstruct gut microbiota balance. Of note, FMT has a more long-term effect on gut microbiota than the ingestion of probiotics, probably because FMT can provide patients with core bacterial species of a healthy population and replace the bacterial components lost in patients, while orally administered probiotics fail to colonize the gastrointestinal tract [90]. For the underlying mechanisms of FMT in the treatment of AD, FMT is reported to modulate the abundance of gut microbiota, the levels of SCFAs, and the immune balance of Th1/Th2 cells in AD mice, mainly manifested as downregulation of Th2 cytokines (IL-4, IL-5, and IL-13) and Treg cytokines (IL-10 and IL-1β), upregulation of Th1 cytokines (IL-12, IFN-γ, and TNF-α), as well as elevation of butyrate, propionate, and acetate [91]. A latest clinical trial evaluating the efficacy of FMT in adult patients with AD has shown that the SCORAD score of AD patients is decreased significantly after FMT [92]. All these suggest that FMT may be a safe and effective treatment intervention for AD patients. Of course, further research is warranted to unveil its specific mechanism.

### 5.4 Addition of essential fatty acids (EFAs)

EFAs include PUFAs (ω-3 and ω-6), linoleic acid (LA), and alpha-linolenic acid (ALA). LA is a member of the ω-6 family, while ALA is classified into the ω-3 family. Studies have shown that LA levels in AD patients typically increase, while their metabolite linolenic acid (DGLA) decreases [93]. As mentioned earlier, the colonization of Malassezia on the skin can trigger inflammatory reactions by producing various enzymes (including lipase and phospholipase) and releasing PUFA. Unsaturated fatty acids have antibacterial effects, thus preventing excessive growth of pathogenic bacteria in the gastrointestinal tract [94]. This suggests that fatty acids may regulate microbial metabolism and mediate the occurrence of AD. Currently, the main focus is on supplementing ω-3PUFAs and ω-6PUFAs safe and biologically active fatty acids to improve clinical symptoms of moderate to severe AD [95]. ω-3PUFAs have powerful anti-inflammatory functions, they can inhibit lymphocyte proliferation, cytokine production, adhesion molecule expression, and trigger cell apoptosis, which can be used to treat the most common chronic inflammatory skin diseases, such as AD, psoriasis, and acne [96]. ω-3PUFAs may also affect the bacterial diversity of the gut microbiota, creating a less inflammatory environment for the colon mucosa during human health and disease treatment [97].

In a word, PUFAs and their derivatives play an important role in the prevention or treatment of AD. The possible mechanisms of PUFAs in treating AD include promoting the differentiation and maturation of the stratum corneum, repairing skin barrier function, reducing the production of cytokines, inhibiting mast cell degranulation, and regulating immunity.

## 6 Summary

This review focuses on the possible mechanisms of the microbiota–skin–gut axis in the pathogenesis of AD and explores new treatment approaches for AD. The therapeutic advantages and potential mechanisms of probiotics, AMPs, microbiota transplantation, and fatty acids are discussed. Among them, probiotics and FMT can not only modulate gut microbiota, but also secrete a variety of metabolites and neurotransmitters to induce the production of anti-inflammatory factors, thereby modulating the immune status of the skin. However, whether supplementation with intestinal probiotics affects AD skin microbiota still warrants further investigation. The addition of AMPs and cons transplantation can reduce *S. aureus* colonization through antagonistic competition against microbiota and affect the innate immune system to restore the immune balance in AD skin.

The microbiota has the ability to function as a signaling molecule, facilitating communication between the skin and gut and thereby impacting the overall balance of the skin–gut axis. This influence extends to several components of the skin–gut axis, including the epidermal barrier, immune system, metabolism, and neuroendocrine system. Based on the role of serum 25(OH)D, UVB, and EFAs in AD, it can be inferred that alterations in skin microbiota may affect the intestinal immune system and thus remodel gut microbiota, which confers novel insights into the possible communication of the skin–gut axis. So far, the underlying mechanisms of the microbiota–skin–gut axis in the treatment of AD have not been thoroughly elucidated. Therefore, we still need to integrate multi-omics, such as meta-transcriptomics, metagenomics, and metabolomics, to provide a new perspective for targeting microbiota in the treatment of allergic skin inflammation.
